# A High-Density EST-SSR-Based Genetic Map and QTL Analysis of Dwarf Trait in *Cucurbita pepo* L.

**DOI:** 10.3390/ijms19103140

**Published:** 2018-10-12

**Authors:** Chenggang Xiang, Ying Duan, Hongbo Li, Wei Ma, Sanwen Huang, Xiaolei Sui, Zhonghua Zhang, Changlin Wang

**Affiliations:** 1 Beijing Key Laboratory of Growth and Development Regulation for Protected Vegetable Crops, College of Horticulture, China Agricultural University, Beijing 100193, China; z0501080535@126.com; 2 Institute of Vegetables and Flowers, Chinese Academy of Agricultural Sciences, Key Laboratory of Biology and Genetic Improvement of Horticultural Crops of Ministry of Agriculture, Sino-Dutch Joint Lab of Horticultural Genomics, Beijing 100081, China; duanying@caas.cn (Y.D.); lihongbo_solab@163.com (H.L.); 15930279531@163.com (W.M.); 3 Agricultural Genome Institute at Shenzhen, Chinese Academy of Agricultural Sciences, Shenzhen 518120, China; huangsanwen@caas.cn

**Keywords:** *Cucurbita pepo*, genetic map, EST-SSR, quantitative trait locus (QTL), dwarf, gibberellin (GA), tetraploid

## Abstract

As one of the earliest domesticated species, *Cucurbita pepo* (including squash and pumpkin) is rich in phenotypic polymorphism and has huge economic value. In this research, using 1660 expressed sequence tags-simple sequence repeats (EST-SSRs) and 632 genomic simple sequence repeats (gSSRs), we constructed the highest-density EST-SSR-based genetic map in *Cucurbita* genus, which spanned 2199.1 cM in total and harbored 623 loci distributed in 20 linkage groups. Using this map as a bridge, the two previous gSSR maps were integrated by common gSSRs and the corresponding relationships around chromosomes in three sets of genomes were also collated. Meanwhile, one large segmental inversion that existed between our map and the *C. pepo* genome was detected. Furthermore, three Quantitative Trait Loci (QTLs) of the dwarf trait (gibberellin-sensitive dwarf type) in *C. pepo* were located, and the candidate region that covered the major QTL spanned 1.39 Mb, which harbored a predicted gibberellin 2-β-oxidase gene. Considering the rich phenotypic polymorphism, the important economic value in the Cucurbita genus species and several advantages of the SSR marker were identified; thus, this high-density EST-SSR-based genetic map will be useful in Pumpkin and Squash breeding work in the future.

## 1. Introduction

The genus *Cucurbita* (2*n* = 2*x* = 40) is perhaps the most polymorphic genus in fruit characters in the botanical family Cucurbitaceae [1]. At least five species of *Cucurbita* have been domesticated by now and three of them, *C. pepo* L., *C. moschata* Duchesne, and *C. maxima* Duchesne, are cultivated world wild [2,3]. Among the many species in the *Cucurbita* genus, *C. pepo* has the most important economic status and a wide range of uses. Besides the fact that the mature and immature fruit, seed, seed oil, and seed extract of *C. pepo* have a great market, its flowers can also be used as food in some places [3,4,5].

Dwarf plant architecture is important for improving agricultural efficiency and reducing the agricultural production costs of modern farming [6,7,8]. *C. pepo* and *C. maxima* are two of the few commercial species with a dwarf plant architecture in the Cucurbitaceae family. Some species with greater economic value in vegetable farming, such as cucumber and melon, have plant architectures that sprawl in vegetable farming, result in massive labor consumption through the growth process. Although some mutants have been reported with a dwarf phenotype in crops with greater economic value, such as cucumber and melon, these dwarf mutants are often accompanied by malformation, which limits the application of dwarf breeding [9,10]. Revealing the dwarf molecular mechanism in *C. pepo* and *C. maxima* will be useful in dwarf breeding and other economically viable species such as cucumber and melon.

Previously, Zhang et al. [11] recovered three dwarf Quantitative Trait Loci (QTLs) in *C.maxima* using genotyping by sequencing (GBS) in an F_2_ population (182 individuals), and suggested the main QTL *qCmB2*, which explains the 21.4% phenotypic variation, was a gibberellin synthesis gene called *GA20ox*. Using two *C. pepo* cultival as the parental line (Zucchini MU-CU-16 and Scallop UPV-196), Esteras et al. [12] and Montero-Pau et al. [13] constructed two single nucleotide polymorphism (SNP)-based genetic maps successively. However, due to there being no significant difference in the plant height trait (bushy versus intermediate) between the two parental lines, no significant QTLs related to the plant height trait were detected in either map. Gong et al. [14] developed 500 genomic simple sequence repeat (SSR) markers and constructed an SSR genetic map in which *Bush* (*B*) for the bush growth habit was located in LGp12, with 7.8 cM from SSR marker CMTp131. However, the relationship between these two species and a detailed molecular mechanism of the dwarf trait are still unknown, although the dwarf trait is important and dominant in *C. maxima* and *C. pepo*.

High-density genetic maps based on molecular markers are widely used in mining advantageous agricultural gene traits, assembling scaffolds in the genome sequencing program and revealing the evolution of the genome [15,16,17]. Research is stalled due to a lack of transferable and co-dominant molecular markers in most species without the reference genome data. Develop expressed sequence tags (EST-SSR) and EST-SNP from transcriptome data provided a method for obtaining useful molecular markers in a short time [18,19]. Compared with gSSR, EST-SSR has the advantage of lower costs in SSR marker development, but with the disadvantage of lower polymorphic potential in genotyping around species [20,21]. However, compared with SNP, EST-SSR has greater polymorphic potential in the single position because EST-SSR relies on length polymorphism in polymerase chain reaction (PCR) products, which makes it more suitable for diversity analysis and fingerprinting [7,22,23]. Gong et al. developed 500 gSSRs using a single-strand probe hybridization method, constructing two genetic maps of *C. pepo* and *C. moschata* ten years ago. These gSSRs were also used in genetic relationship analysis in *C. pepo* [14,24,25]. In early research, Ren et al. [26] developed a large number of gSSRs from the cucumber genome and identified 132 gSSRs with PCR products in *C. moschata*. Blanca et al. [27] developed a large number of EST-SNP and EST-SSR from the transcriptome data in *C. pepo*. Later, Esteras et al. [12] reported an EST-SNP and gSSR mixed map developed by Gong et al. [14,24], but only 11 gSSRs were included in that map, which made it difficult to integrate the two previous gSSR maps [12,26,27].

Three recently published sets of genome data (*C. maxima*, *C. moschata* and *C. pepo*) and three high density genetic maps (both SNP-based, GBS or dd-RAD sequencing) will facilitate the mining of advantageous agricultural traits in the *Cucurbita* genus [11,13,28,29,30]. However, the collinearity around each chromosome between *C. pepo* and *C. maxima*/*C. moschata* are still unknown; furthermore, all of these published genetic maps are based on SNP markers, which relied on the sequencing data, leading to a greater cost when validating the markers in other species of the *Cucurbita* genus.

Considering the species in the *Cucurbita* genus with a rich polymorphism, a large number of advantageous agricultural traits, and a high-density genetic map could integrate previous and current work. In this research, using an F_2_ population (187 individuals) derived from two parents with huge phenotypic differences (Figure S1) and 2245 SSR markers (including 1613 EST-SSRs developed from the transcriptome data of *C. pepo*, 500 gSSRs developed from pumpkin and 132 gSSRs from cucumber), we constructed a high-density EST-SSR-based genetic map in *C. pepo*. The collinearity with the three published genomes and the two gSSR maps in the *Cucurbita* genus were also evaluated. The QTLs of the dwarf trait both at the young and mature developmental stages were also located. By analyzing the synteny with cucumber, melon, and watermelon genomes, the ancient tetraploid origin in *Cucurbita* was proven again.

## 2. Results

### 2.1. A High-Density SSR-Based Genetic Map

In order to augment the number of SSR markers in *C. pepo*, we picked up 1613 novel EST-SSR loci from transcriptome data (Data S1). In combination with 632 previously reported gSSRs [14,26], we then tested the polymorphism of 2245 SSRs between the parents of the F_2_ population used in this study, revealing a total of 1559 SSRs in both parents that yielded PCR products. Of these, 646 exhibited polymorphism between parents, including 133 previously reported gSSRs and 513 EST-SSRs (Data S1).

**Figure 1:**
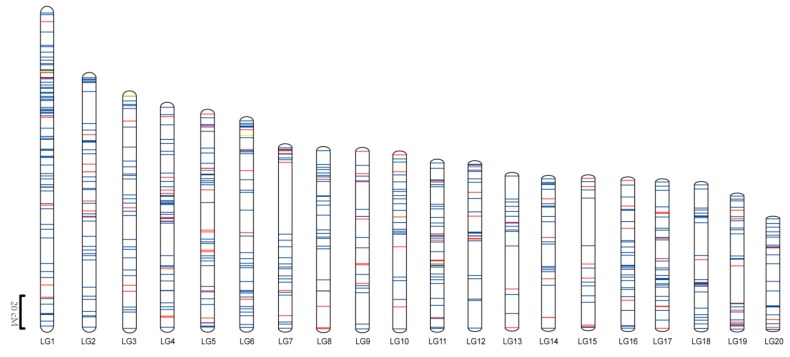
The linkage map of *C. pepo* constructed in this research. Blue, red, and yellow bars indicated the EST-SSR marker, gSSR marker developed from *Cucurbita* and gSSR marker developed from the cucumber genome, respectively. The scaleplate on the left indicated genetic distance (centimorgan as unit, abbreviated cM). LGs represented linkage groups, from LG1 to LG20.

Following the removal of the ambiguous markers, 623 polymorphic SSR markers remained, including 127 gSSRs and 496 EST-SSRs (Table S1). These were then plotted onto 20 LGs to produce a genetic map that encompasses 2199.1 cM and is estimated to cover 93.3% of *Ge* (expected genome length) (2356.3 cM). Lengths of the 20 LGs range between 67.35 cM in LG20 and 190.97 cM in LG1, while marker numbers range from 16 in LG13 and LG15 to 74 in LG1 (Table 1, Figure 1 and Data S2).

### 2.2. Collinearity Analysis

To integrate two gSSR maps constructed by Gong et al. [14,24], we counted the common gSSR markers located in our map and two previous maps. In total, 74 common markers detected with previous *C. pepo* map, distributed in 20 previous linkage groups (20LGp in total); meanwhile 62 common markers detected with a previous *C. moschata* map were distributed in 23 previous linkage groups (27 LGm in total) (Data S2). Overall, the map constructed in this research showed good collinearity with the two previous maps; most of the linkage groups showed a consistent marker order, such as LGp16 vs. LG4 and LGm1a vs. LG2. A deranged marker order was observed between a few linkage groups, such as LGp3a vs. LG9 and LGm3 vs. LG4 (Figure S2). Some separated linkage groups, such as LGp5 and LGp2, LGm21a and LGm21b, were integrated into one linkage group according to the common markers within our genetic map. Some unique linkage groups, such as LGp10a, LGp15, LGp3a and LGp9 with common gSSRs were distributed in different linkage groups during this research (Figure S2).

To validate the accuracy of this map, and collate the respondence relationships of the chromosomes between published genomes, the unigene sequences of EST-SSRs located in this map were aligned to three sets of published *Cucurbita* genomes using tBLASTx [13,29]. In total, 496 EST-SSR were located in our map: 469 (94.56%), 475 (95.77%), and 468 (94.35%) hit the *C. pepo*, *C. moschata* and *C. maxima* genomes, respectively (Table S2).

**Figure 2:**
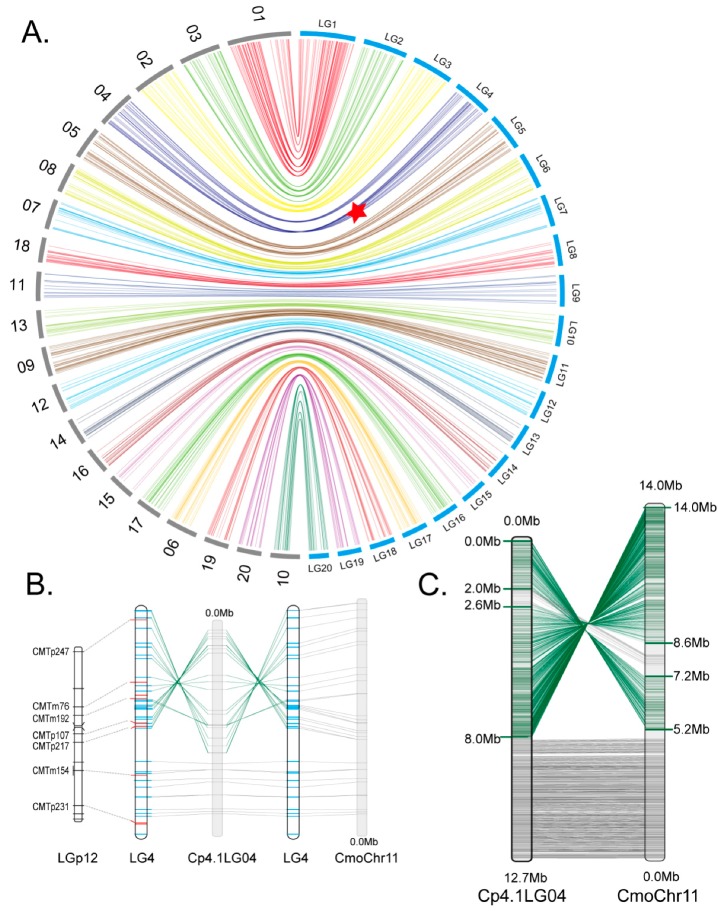
Collinearity between genetic map and physic map of *C. pepo*. (**A**) Collinearity between genetic map (blue) and physic map (gray) of *C. pepo*. The inversion existed in between LG4 and Cp4.1LG04 was indicated by a red pentagram; (**B**) collinearity between LG4 and LGp12, LG4 and Cp4.1LG04 (*C. pepo* genome), LG4 and CmoChr11 (*C. moschata* genome). The common gSSRs between LG4 and LGp12 were represented by grey broken lines. The in order hits between LG4 and Cp4.1LG04, CmoChr11 were represented by grey line and disorder hits were represented by the dark green line. EST-SSRs are represented by the blue bars and gSSRs by red bars in LG4; (**C**) collinearity between Cp4.1LG04 and CmoChr11. In order and disorder hits were represented by grey and dark green lines respectively. The disorder regions were marked according to the alignment result.

In general, the loci located in our map consisted of their corresponding location in the *C. pepo* physic map, which suggests the high quality of our genetic map (Figure 2A). This map also showed high collinearity with the physic map of *C. moschata* and *C. maxima* (Figure S3), especially the *C. moschata* map, which suggested that a high karyotype stability also existed between *C. pepo* and *C. moschata*, not only between *C. moschata* and *C. maxima* [29]. All linkage groups showed good coverage, with three physic maps except LG8, LG17, and LG20 with less than 70% coverage (Table S2). Using our map as a bridge, the correspondence relationship of the chromosomes between *C. pepo* and *C. moschata*/*C. maxima* were also collated (Table S2).

Unexpectedly, one large deviation that was observed in the alignment between LG4 and Cp4.1LG04 of the *C. pepo* genome but not in alignment between LG4 and Chr11 of *C. moschata* genome (Figure 2B). Furthermore, LG4 showed very good collinearity with LGp12 (developed by Gong et al. 2008a) (Figure 2B), which suggested a specific chromosome inversion existed in Cp4.1LG04. To obtain more detailed information of this inversion, all CDS (1629 in total) predicted in Cp4.1LG04 of the *C. pepo* genome were aligned to the *C. moschata* genome by tBLASTx. Totally, 1617 hits to Chr11 of *C. moschata*, in this, CpLG04 showed very good collinearity to Chr11 of *C. moschata* from 2.0 Mb to 2.6 Mb and from 8.0 Mb to 12.7 Mb. The physic region of Cp4.1LG04 from 0.0 Mb to 2.0 Mb and from 2.6 Mb to 8.0 Mb shows two entire inversions corresponding from 7.2 Mb to 5.2 Mb and from 8.6 Mb to 14.0 Mb in Chr11 of *C. moschata* (Figure 2C).

### 2.3. QTLs for Dwarf Trait

Plant height depends on the total number of nodes and the length of each internode in a stem. In the *Cucurbita* genus, early research showed changing dominant-recessive relationships between the young and mature stages [11,31,32]. To get detailed QTL information of a dwarf trait, we investigated the nodes number and internode length of the two parental lines and their hybrid (P_1_/P_2_/F_1_) at the young and mature developmental stages, respectively, in three independent environments. No significant difference was found in the number of nodes between the two parental lines at the young and mature stages (Figure S4A). Conversely, a significant difference was found in internode length between the two parental lines at both the young and mature stages, suggesting that the dwarf trait was controlled by the internode length rather than the internode number in *C. pepo* (Figure S4B).

In *C. maxima* the internode length of the hybrid was similar to the dwarf parent at the young stage and with intermediate value at the mature stage [11]. This developmental reversal was also found in our research (Figure S4B). The average value of internode length in F_2_ population skewed toward the dwarf parent at a young stage and toward the hybrid at the mature stage. The frequency distribution among the test lines suggested that the dwarf was dominant to vein at the young stage because no significant segregation was observed in BC_1_P_2_ (Tables S3 and S4).

**Figure 3:**
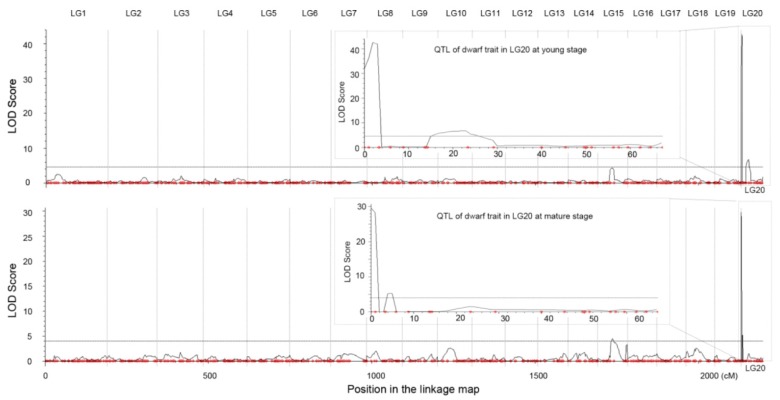
Mapping of quantitative trait loci (QTLs) controlling dwarf traits in the young (**top**) and mature (**bottom**) developmental stages. Curve in plot indicates the LOD score. The boxes inside showed the zoom-in view of the peak on LG20. The molecular markers located in the genetic map were represented by red dots.

Using joint analysis (LOD ≥ 4.0), two and three QTLs associated with dwarf traits at the young and mature stages were detected respectively (Figure 3). In this, the two most significant QTLs (*qCpDy1* and *qCpDm1*) at different developmental stages shared similar candidate regions and LOD values, which spanned a genetic distance with 3.37 cM (from PU051973 to end of LG20) corresponded to a physical distance of 1.39 Mb. This region was also similar to the major dwarf QTL region *qCmB2* in *C. maxima* (Figure S4C and Table S5).

The early research in *C. maxima* found that dwarf line SQ026 was gibberellin-sensitive, in which the internode length could be rescued to the vein parental internode length after being treated with exogenous gibberellin (GA). To confirm if the similar mechanism existed in *C. pepo*, the dwarf parent HM-S2 in this research was treated with gradient exogenous GA and the vein parent Jinganlu was treated with gradient Paclobutrazol (PAC, GA synthesis inhibitor). The results of the exogenous hormone treatment showed the dwarf type in *C. pepo* was also a GA-sensitive dwarf type (Figure S4D).

Although several experimental observations in *C. pepo* are similar with the previous results in *C. maxima* (F_1_ hybrid developmental reversal, major QTL position, and gibberellin sensitivity), *qCmB2*, which designated as a *GA20ox* controlling dwarf trait in previous research, was denied by HMMER prediction. Only one *GA2ox* and one *GA20ox* detected in Cp4.1LG12 of *C. pepo* genome and in Chr03 of *C. maxima* genome, respectively, and only one *GA2ox* gene related to the gibberellin synthesis in the candidate region (Figure S4C).

### 2.4. Ancient Tetraploid Origin

**Figure 4:**
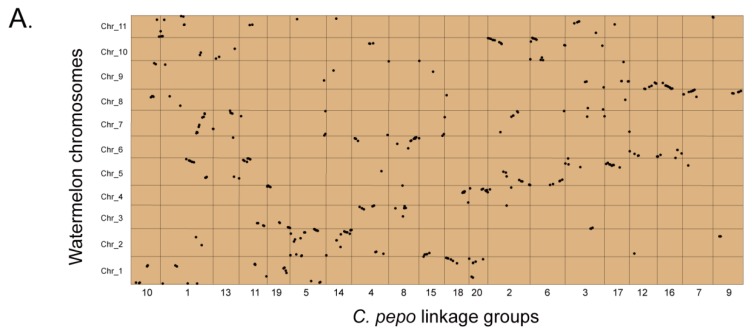

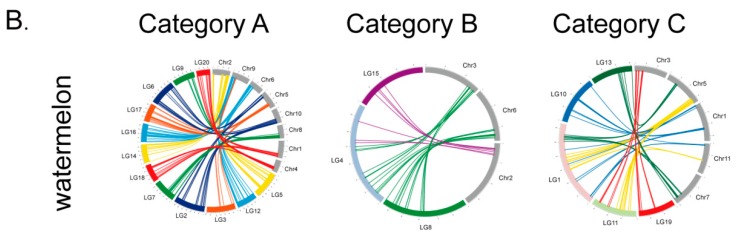
Alignment result with watermelon genome and the pairwise relationship around linkage groups. (**A**) Dot plots represent the alignment results between *C. pepo* and the watermelon genomes. The horizontal axis shows the genetic position of 20 *C. pepo* LGs, while the vertical axis shows the physical position of the 11 watermelon chromosomes. Each dot corresponds to a single marker and represents one tBLASTx hit between *C. pepo* LGs and watermelon chromosomes; (**B**) circos plots that reveal the presence of three categories on the basis of syntenic relationships between *C. pepo* and watermelon. The LGs in this figure are shown with colored bars while the chromosomes of watermelon are shown with grey bars. The outside scale bars refer to cM in the case of LGs and Mb in the case of chromosomes, respectively. Category A includes twelve LGs (LG20 and LG18, LG5 and LG14, LG16 and LG12, LG17 and LG3, LG2 and LG6, and LG7 and LG9) that show roughly two-to-one relationships with chromosomes of watermelon, melon, and cucumber. Category B includes three LGs (LG4, LG8, and LG15). The front region of LG4 corresponds with LG8 while the backend region of LG4 corresponds to LG15. No syntenic region is shared between LG8 and LG15 in the three genomes. Category C includes five LGs (LG1, LG10, LG11, LG13, and LG19). The backend region of LG11 corresponds to LG19 while the front region of LG11 corresponds to the middle region of LG1. The front region of LG1 corresponds to LG10 and the backend region of LG1 corresponds to LG13.

Sequence order conservation (synteny) provides the basis for the transfer of genomic information between species. Thus, to determine if *C. pepo* experienced an additional whole genome duplication (WGD) event after the divergence from the *Bennicase* tribe, we aligned an unigene sequence from the 496 EST-SSR markers in our genetic map against the watermelon, melon, and cucumber genomes using tBLASTx. In total, 374, 315, and 340 hits were identified according to the alignment result with the watermelon, melon, and cucumber genomes respectively. In general, the alignment results showed a pairwise relationship existing around 20 linkage groups, for example, a large number of loci in LG7 and LG9 with hits in Chr8 of the watermelon genome, and a large number of loci in LG2 and LG6 with hits in Chr5 and Chr10 (Figure 4A). These pairwise relationships were also confirmed by the alignment results with the melon and cucumber genomes (Figure S5A and S5B). After removing the ambiguous hits, finally, 282 hits (75.4% in 374) with watermelon genome (Figure 4B), 264 hits (83.8% in 315) with the melon genome (Figure S5C), and 284 hits (83.5% in 340) with the cucumber genome (Figure S5D) showed distinguishable evidence for the tetraploid origin in *C. pepo*.

Generally, 20 *C. pepo* LGs can be classified into three categories based on their syntenic relationships with watermelon, melon, and cucumber. Among these, category A includes 12 LGs (LG20 and LG18, LG5 and LG14, LG16 and LG12, LG17 and LG3, LG2 and LG6, and LG7 and LG9) that exhibit approximate two-to-one relationships with chromosomes in the three sequenced genomes. Similarly, category B comprises three LGs (LG4, LG8, and LG15), of these, the front part region of LG4 showed pairwise relationship with LG8 and the back part region of LG4 showed a pairwise relationship with LG15. Category C includes the remaining five LGs (LG1, LG10, LG11, LG13, and LG19), the front part region of LG1 showed a pairwise relationship with LG10, the middle part region of LG1 showed a pairwise relationship with the front part region of LG11, in which the end part region showed a pairwise relationship with LG19, the end part region of LG1 showed a pairwise relationship with LG13 (Figure 4B and Figure S5C,D).

Because of a lack of enough EST-SSR loci in our genetic map, some small segments with a pairwise relationship reported in a previous genome sequencing report could not be detected in this research, however, the relationships conducted by our data roughly coincided with our genome sequencing results. For example, Cma08 and Cma17, Cma03 and Cma07, which showed a typical pairwise relationship, were also proven in our research (LG16 and LG12, LG20 and LG18, respectively). These results suggested that the high-density EST-SSR-based map could be used in an ancient polyploid event, roughly discovering and decrypting the chromosomes evolutionary relationship.

## 3. Discussion

### 3.1. EST-SSR with Higher Polymorphism than gSSR

Compared with SNP, SSR had higher polymorphic potential and lower cost (not relying on sequencing data) [7,22,23]. In *Cucurbita* genus, four SNP-based genetic maps have been published in recent years, but only two SSR-based genetic maps published ten years ago and with the disadvantage of a lower marker density. In this research, using 634 gSSRs and 1613 novel EST-SSRs, we constructed the first high-density EST-SSR-based genetic map in *Cucurbita* genus, the total mapped loci number in this map was lower than one of the SNP maps in *C. pepo* (GBS, RILs population) and one SNP map in *C. moschata* (dd-RAD, F_2_ population) but higher than the two SNP maps in *C. pepo* (EST-SNP, F_2_ population) and *C. maxima* (GBS, F_2_ population) [11,12,13,30]. According to common gSSR information and alignment results with published genome data, the other gSSRs located in the previous two gSSR maps but not in this map could also be integrated with the corresponding chromosome. Considering the rich phenotypic polymorphism in *Cucurbita* genus, these gSSR and EST-SSR markers will be useful in constructing molecular fingerprintings and primary mapping of the agronomic traits.

In general, as EST-SSRs are derived from transcribed genomic regions, they could be transferred across various species but with a lower polymorphic potential, compared with gSSR [15,33]. However, in this research, EST-SSR showed higher polymorphism than gSSR (31.8% versus 24.5%) (Table S1), and these differences are likely because of the selection strategy in the transcriptome data. We preferred to employ EST-SSR with higher A/T content motifs while at the same time avoiding those with higher G/C content motifs because these potentially exhibit lower levels of polymorphism [34,35].

### 3.2. Collinearity Analysis Reveals an Inversion Existed Between Our Map and Ref Genome

In *Cucurbita* genus, three sets of genome data were published in the last year [28,29], however, the corresponding relationship between chromosomes in *C. pepo* and in *C. maxima*/*C. moschata* is still unknown. According to the alignment result between the genetic map and the three genomes of *Cucurbita*, we collated the correspondence relationships of each of the chromosomes. Overall, the genetic map showed good collinearity with three published genomes, which consisted of the previous conclusion about karyotype stability in *Cucurbita* [29]. The collinearity with *C. moschata* is better than with *C. maxima*, this could be explained by *C. pepo* with a closer relationship with *C. moschata*, which was proven in previous research [3,36].

Theoretically, this map should show the best collinearity with the *C. pepo* genome but not with *C. moschata*, this unexpected result was due to one large segmental inversion detected in LG4 vs. CpLG04. However, we are not sure whether the chromosomal inversion in LG4 detected in this research actually exists on the true *C. pepo* genome.

On the one hand, although the mapping parental lines (both *C. pepo* subsp. *pepo*, zucchini and pumpkin cultival-type) used in this research are different than the parental lines (*C. pepo* subsp. *pepo* vs. *C. pepo* subsp. *ovifera*, zucchini and scallop cultival-type) used in the genome sequencing program, inter-subspecific crosses (*C. pepo.* subsp*. pepo* vs. *C. pepo.* subsp*. ovifera*, pumpkin and crockneck cultival-type) were also used in Gong’s research, which give a good collinearity between LG4 and LGp16 (Figure 2B). On the other hand, chromosome inversions that occur on the chromosomes often lead to infertility in offspring [37,38,39]; however, no distinguishable productive isolation was found in our breeding work around zucchini, scallop, crockneck, and pumpkin cultival-types lines and their hybrids, which makes us even more skeptical about whether this chromosome inversion really exists.

Given the important economic value of some species in *C. pepo* and the fundamental role of the genome sequence data in the mining gene related to the agronomic trait, we suggested that some of the following experiments still may be needed, such as fluorescent in situ hybridization (FISH) and high density genetic maps using other parental lines, to get a definitive conclusion.

### 3.3. Dwarf Trait in C. maxima and C. pepo Perhaps Controlled by Same Gene

In this research, the candidate region of the dwarf trait was mapped to the *C. pepo* genome for the first time, and the linkage group which harbored the dwarf gene (LG20) was the same as Gong et al.’s work [14], in which the *Bush* gene (*B*) mapped to LGp12; however, the major dwarf gene in our map and the *Bush* gene in Gong et al.’s map [14] were not in a common candidate region (Figure S4C). Considering only two SSRs, located in LGp12 (CMTm61 and CMTp36) in Gong et al.’s map [14], we suggested the gSSR and EST-SSR markers located in LG20 could be used in Gong et al.’s population [14]. After getting a more accurate candidate region and then comparing the results from this experiment, we confirmed whether the dwarf gene shared a common region around different parental lines in *C. pepo*.

The characters of the dwarf trait observed in this research showed a large similarity with the characters reported in Zhang et al.’s work [11] (developmental reverse in hybrid, major QTL candidate region, and hormone response), however, the LOD value of major dwarf QTL in both *qCpDy1* and *qCpDm1* were much higher than *qCmB2*, and the other two dwarf QTLs (*qCmB1* and *qCmB3*) reported in *C. maxima* were not detected in *C pepo*. These differences suggested that the molecular mechanism control dwarf trait in *C. pepo* were not exactly the same as in *C. maxima*.

For the phenomenon of developmental reversal, Zhang et al. [11] proposed that there were other genes involved in a plant grown to the mature stage in *C. maxima*. However, in *C. pepo*, the major dwarf QTL at the young and mature developmental stages shared a common region and both had a large LOD value (Figure 3 and Table S5), which suggested the dwarf and reversal phenomenon were perhaps controlled by the same gene, and the developmental reversal was due to different expression value of this gene at different developmental stages.

The major dwarf QTL *qCmB2* in *C. maxima* was designated as a *GA20ox* in Zhang et al.’s work [11], and the dwarf phenomenon was explained by a lower expression level in the dwarf parent. However, this in not supported by HMMER prediction in which only a *GA2ox* gene exists in the candidate region (Figure S4C). Furthermore, a lower *GA20ox* expression level seems hard to explain why the internode length of a hybrid to a dwarf parent is especially close at a young stage. Based on the dosage effect, the hybrid should be with intermediate or similar internode length to vein parental, because half of the *GA20ox* gene was still expressed normally in the hybrid. Research related to the *GA20ox* mutant has shown the vein was dominant or semi-dominant to the dwarf [40,41,42], but research in *C. maxima* and *C. pepo* suggested that the dwarf was dominant to vein [11,32,43]. These results suggested the the dwarf molecular mechanism in *Cucurbita* could not be explained simply by a depressed expression level in *GA20ox*.

Although one *GA2ox* was detected in the candidate region, due to lacking enough molecular markers in candidate region, we are not sure if this *GA2ox* gene is the gene that controls the dwarf phenotype, because there are also a large number of reports about transcription factors that control dwarf traits by regulating the expression of a GA synthesis gene [44,45,46]. To get a more detailed explanation about the dwarf molecular mechanism in *C. pepo*, we will develop more markers from the resequencing data of the two parental lines used in this research and employ a larger F_2_ population in the future.

### 3.4. EST-SSR-Based Genetic Map Could Be Useful in Revealing Ancient WGD Event

Based on the alignment results in this research, it is difficult to determine whether the ancient polyploid event happened in *C. pepo* belongs to an Auto-ploid type or an Allo-ploid type for lacking large amounts of sequencing data information [47]. However, we discovered strong evidence of the tetraploid origin in *C. pepo* and collated pairwise linkage groups from the alignment results with genomes of adjacent species, which is consistent with the conclusion analyzed from the genome *De novo* sequencing data [28,29]. Considering that whole genome *De novo* sequencing is still expensive for most species without a reference genome, and the ancient polyploid event happened frequently [48,49], we suggested that it is still an efficient and lower cost method to research chromosomal evolution through developing EST-SSR or EST-SNP from the transcriptome data and constructing a high density genetic map to align with known genomes [16,50,51].

## 4. Materials and Methods

### 4.1. Plant Materials and Phenotyping

Two inbred lines differing in several phenotypes were used as the mapping parents for this study (Figure S1). Of these, the P_1_ (Jinganlu) belongs to the *C. pepo* subsp. *pepo* var. Pumpkin, while the P_2_ (HM-S2) belongs to *C. pepo* subsp. *pepo* var. Zucchini. Phenotypic evaluations were carried out three times for the replicates and SPSS 17.0 was used to perform the phenotypic data analysis. One six-generation population (P_1_/P_2_/F_1_/BC_1_/BC_2_/F_2_) was grown at the CAAS experimental station (N39.56°, E118.18°) in the autumn of 2012 (BJA). Plant height, internode number and internode length of two parental lines, hybrid, F_2_ population and BC population were investigated at young (25 days after sowing) and mature (60 days after sowing) developmental stages, respectively. To validate the developmental reversal phenomenon in a hybrid and dominant-recessive relationship in the segregating population, two parental lines and their hybrid (P_1_/P_2_/F_1_) were grown at the langfang experimental station (N39.36°, E116.36°) in the autumn of 2014 (LFA) and one four-generation population (P_1_/P_2_/F_1_/F_2_) was grown at the langfang experimental station (N39.36°, E116.36°) in the summer of 2015 (LFS), investigating the plant height, internode number and internode length at the young and mature developmental stages, respectively.

### 4.2. SSR Search

A total of 2245 SSR markers were utilized in this study, including 132 gSSRs from the cucumber genome, isolated with PCR products for *C. moschata* [26], and 500 gSSR (specifically, 307 from *C. moschata* and 193 from *C. pepo*) as developed by Gong et al. [14] using single-stranded hybridization. A further 1613 EST-SSRs were also developed for this study using transcriptome data for *C. pepo* (http://www.icugi.org). All the primers were synthesized by Sangon Biotech (Sangon, Shanghai, China).

### 4.3. DNA Extraction and PCR Amplification

Genomic DNA from young leaves was extracted using the CTAB method with minor modifications. DNA quality was assessed using 1.2% agarose gel, and quantity was assessed using NanoDrop 2000 Spectrophotometer (Thermo Scientific, Waltham, MA, USA). A polymerase chain reaction (PCR) was carried out on Bio-Rad T100 thermal cycler (Bio-Rad, Hercules, CA, USA). PCR reactions were prepared in 12.5 μL volumes containing 10 × Taq polymerase buffer (500 mM KCl, 100 mM Tris-HCI pH 8.5, and 1 mg/mL gelatin), 1.0 mM MgCl_2_, 0.5 mM dNTPs, 5 pM of each primer, 0.3 U Taq polymerase, and 25 ng of template DNA, respectively. The final volume was adjusted to 12.5 μL with sterile distilled water, and PCR amplifications were set at 95 °C for 10 min, followed by 35 cycles at 94 °C for 1 min and 20 s at a specific annealing temperature for a specific primer pair and then at 74 °C for 30 s, before final extension at 74 °C for 10 min. All PCR products were separated on a 6% non-denaturing PAGE electrophoresis system and were visualized using silver staining [52].

### 4.4. Genotyping and Map Construction

Both parents and their F_1_ offspring were used to select polymorphic markers; markers that had product lengths ranging between 80 bp and 300 bp and that also showed polymorphism between parents were used to construct the linkage map. A total of 187 individuals from the F_2_ population (BJA) were used to generate the linkage map. Segregation distortion at each marker locus was tested against the expected ratio for F_2_ (1:2:1) using a Chi-square test. Those markers that showed highly significant segregation distortion (*p* < 0.01) were excluded from the map construction. Linkage analysis was then performed using the regression function in the software JoinMap v4.1. Thus, linkage groups (LGs) were determined using a LOD threshold of 4.0 with the Kosambi mapping function used for linkage analysis. The parameters for this analysis were rec = 0.4, LOD = 1.0, Jump = 5.0, and markers that had linkage lengths that were too long were excluded (>50 cM). The observed genome length (*Go*) was calculated by summing the observed map length of all the linkages. The expected length of each LG was then estimated using method of Chakravarti et al. [53] and expected genome length (*Ge*) was then estimated by summing the lengths of the estimated LGs. The observed genome map coverage (*GCo*) was defined as the ratio between the total length of the map *Go* and *Ge* [54].

### 4.5. Collinearity Analysis

Unigene sequences for all EST-SSR loci in the genetic map were aligned against the *C. pepo*, *C. maxima*, *C. moschata*, watermelon, melon, and cucumber genomes using tBLASTx (*E*-value < e^−10^) [55]. For a visual representation of the analysis results, the R package Omiccircos was used to draw the circos-plot [56].

### 4.6. Detection of Dwarf QTLs and Hormone Application

The QTLs of dwarf trait were detected by IciMapping V4.1 (available online: https://www.integratedbreeding.net/386/breeding-services/more-software-tools/icimapping) based on the inclusive composite interval mapping (ICIM) model [57]. The ICIM-ADD method was used to detect additive and dominant QTLs and estimate relative genetic parameters. The threshold value was determined by 1000 permutations and the type I error set as *p* < 0.05, the *PIN* value = 0.001, *step* value = 0.5 cM.

Gibberellin (GA) and Paclobutrazol (PAC) were resolved in ethanol. For the treatments, the chemical reagents were diluted to a desired concentration using water and spray to 25-day-old and 60-day-old plants. The blank solvent (add appropriate ratio ethanol) were used as mock treatment, all treatments were performed twice at intervals of 4 days.

### 4.7. Candidate Gene Analysis

HMMER and BLAST were used to screen the putative gibberellin synthesis genes [58]. The HHM profile of the *GA20ox*, *GA3ox* and *GA2ox* family gene domain was downloaded from the Pfam database (PF03171, https://pfam.xfam.org/family/PF03171) and the peptide sequence data of *C. pepo* and *C. maxima* genomes was downloaded from the ICUGI (http://cucurbitgenomics.org/). The local BLAST program V2.7.1 was used to remove the repetitive sequence of putative *GA20ox*, *GA3ox* and *GA2ox* family gene, and TBtool V0.664 (available online: http://cj-chen.github.io/tbtools/) was used to locate the respective genes on the reference genome.

## Figures and Tables

**Table 1:** Description of the 20 linkage groups (LGs) in *Cucurbita pepo*.

LG	Number of Markers	Marker Density (cM/Marker)	Number of EST-SSR	EST-SSR Percentage (%)	Observed Length (cM)	Expected Length (cM)	Observed Coverage (%)	Largest Gap (cM)
LG1	74	2.58	65	87.8	190.97	196.20	97.33	15.61
LG2	43	3.54	35	81.4	152.13	159.37	95.45	19.53
LG3	32	4.43	27	84.4	141.87	151.02	93.94	14.72
LG4	44	3.05	34	77.3	134.19	140.43	95.56	19.85
LG5	43	3.02	31	72.1	129.92	136.11	95.45	16.74
LG6	36	3.50	30	83.3	126.00	133.20	94.59	13.61
LG7	26	4.23	20	76.9	110.03	118.83	92.59	43.83
LG8	27	4.02	22	81.5	108.60	116.95	92.86	18.92
LG9	20	5.41	13	65.0	108.30	119.69	90.48	19.12
LG10	24	4.42	19	79.2	106.14	115.37	92.00	18.51
LG11	37	2.73	30	81.1	100.95	106.55	94.74	10.77
LG12	25	3.99	18	72.0	99.85	108.17	92.31	21.12
LG13	16	5.78	13	81.3	92.53	104.87	88.24	26.22
LG14	23	3.96	18	78.3	91.16	99.44	91.67	19.50
LG15	16	5.68	10	62.5	90.91	103.03	88.24	29.0
LG16	29	3.11	25	86.2	90.20	96.64	93.33	9.32
LG17	31	2.88	24	77.4	89.26	95.21	93.75	11.30
LG18	27	3.25	23	85.2	87.67	94.41	92.86	20.0
LG19	27	3.00	19	70.4	81.12	87.36	92.86	22.87
LG20	23	2.93	20	87.0	67.35	73.47	91.67	10.84
Mean	31.15	3.78	24.8	78.5	109.96	117.82	93.33	
Total	623		496		2199.10	2356.31		

## Supplementary Materials

Supplementary materials can be found at http://www.mdpi.com/1422-0067/19/10/3140/s1.

## Abbreviations

QTL	Quantitative Trait Locus
GA	Gibberellin Acid
EST	Expressed Sequence Tag
SNP	Single Nucleotide Polymorphisms
SSR	Simple Sequence Repeat
WGD	Whole Genome Duplication
