# Supplementary material for: A High-Density EST-SSR-Based Genetic Map and QTL Analysis of Dwarf Trait in Cucurbita pepo L
Source: Int J Mol Sci. 2018 Oct 12;19(10):3140. doi: 10.3390/ijms19103140 (PMC6213718; doi:10.3390/ijms19103140)
Supplement: Supplementary file 1 [file ijms-19-03140-s001.zip › Supplementary Files/Table S1.DOCX]

**Table S1. SSR selection and genotyping.** Ratios are shown in brackets next to total numbers.

| **Origin** | **Total used** | **With PCR product** | **Polymorphic between parents** | **Map** |
| --- | --- | --- | --- | --- |
| *C. moschata* gSSRs | 307 | 248 (80.78%) | 80 (26.06%) | 80 (26.06%) |
| *C. pepo* gSSRs | 193 | 171 (88.60%) | 49 (24.87%) | 45 (23.32%) |
| Cucumber gSSRs | 132 | 35 (26.50%) | 4 (3.00%) | 4 (3.00%) |
| *C. pepo* EST-SSRs | 1,613 | 1105 (68.50%) | 513 (31.80%) | 496 (30.7%) |
