# Supplementary material for: A High-Density EST-SSR-Based Genetic Map and QTL Analysis of Dwarf Trait in Cucurbita pepo L
Source: Int J Mol Sci. 2018 Oct 12;19(10):3140. doi: 10.3390/ijms19103140 (PMC6213718; doi:10.3390/ijms19103140)
Supplement: Supplementary file 1 [file ijms-19-03140-s001.zip › Supplementary Files/Table S2.DOCX]

**Table S2. Correspondence relationships between linkage groups (LGs) and chromosomes of *C. pepo*, *C. moschata* and *C. maxima* genomes.**

| **Linkage**  **group** | **EST-**  **SSR** | ***C. pepo*** | | | ***C. moschata*** | | | ***C. maxima*** | | |
| --- | --- | --- | --- | --- | --- | --- | --- | --- | --- | --- |
|  |  | **hit** | **Cover length^a^** | **LGp4.1LGs^b^** | **hit** | **Cover length^a^** | **Cmo. Chrs^b^** | **hit** | **Cover length^a^** | **Cma. Chrs^b^** |
| 1 | 65 | 63 | 20.6 (96.7%) | 01 (21.3) | 65 | 21.2 (96.9%) | 04 (21.9) | 60 | 19.7 (99.4%) | 04 (19.8) |
| 2 | 35 | 32 | 13.5 (97.7%) | 03 (13.8) | 35 | 15.6 (98.0%) | 14 (15.9) | 32 | 14.6 (97.9%) | 14 (14.9) |
| 3 | 27 | 25 | 13.6 (94.3%) | 02 (14.4) | 27 | 13.8 (94.3%) | 01 (14.6) | 27 | 12.3 (93.7%) | 01 (13.1) |
| 4 | 34 | 30 | 11.0 (86.6%) | 04 (12.7) | 34 | 12.7 (90.5%) | 11 (14.0) | 31 | 11.7 (89.8%) | 11 (13.0) |
| 5 | 31 | 30 | 10.8 (99.0%) | 05 (10.9) | 31 | 10.4 (99.1%) | 02 (10.5) | 30 | 10.0 (99.2%) | 02 (10.1) |
| 6 | 30 | 28 | 9.9 (98.6%) | 08 (10.1) | 30 | 11.4 (95.3%) | 06 (12.0) | 26 | 10.2 (94.7%) | 06 (10.7) |
| 7 | 20 | 19 | 9.4 (92.5%) | 07 (10.1) | 20 | 11.6 (98.3%) | 12 (11.8) | 19 | 10.0 (98.3%) | 12 (10.2) |
| 8 | 22 | 21 | 5.6 (66.7%) | 18 (8.3) | 22 | 5.7 (43.3%) | 10 (13.1) | 22 | 5.3 (60.0%) | 10 (8.8) |
| 9 | 13 | 13 | 8.2 (83.5%) | 11 (9.8) | 13 | 9.4 (86.1%) | 05 (10.9) | 12 | 9.1 (86.1%) | 05 (10.6) |
| 10 | 19 | 19 | 9.0 (95.7%) | 13 (9.4) | 19 | 10.0 (96.1%) | 15 (10.3) | 19 | 8.8 (95.9%) | 15 (9.2) |
| 11 | 30 | 27 | 9.6 (96.6%) | 09 (9.9) | 30 | 12.6 (97.1%) | 18 (12.9) | 29 | 9.9 (96.7%) | 18 (10.3) |
| 12 | 18 | 18 | 9.0 (91.9%) | 12 (9.8) | 18 | 9.8 (91.0%) | 17 (10.8) | 18 | 8.7 (91.0%) | 17 (9.5) |
| 13 | 13 | 13 | 7.4 (82.7%) | 14 (9.0) | 13 | 7.9 (83.2%) | 16 (9.5) | 13 | 8.2 (83.7%) | 16 (9.8) |
| 14 | 18 | 18 | 6.3 (72.8%) | 16 (8.7) | 18 | 9.1 (77.1%) | 20 (11.8) | 18 | 7.3 (78.2%) | 20 (9.3) |
| 15 | 10 | 9 | 8.4 (95.7%) | 15 (8.8) | 10 | 9.4 (96.4%) | 19 (9.8) | 9 | 9.0 (96.2%) | 19 (9.3) |
| 16 | 25 | 25 | 8.4 (96.7%) | 17 (8.7) | 25 | 7.5 (93.2%) | 08 (8.0) | 23 | 7.4 (92.8%) | 08 (7.9) |
| 17 | 24 | 24 | 7.1 (66.9%) | 06 (10.7) | 24 | 6.8 (55.9%) | 09 (12.2) | 23 | 6.1 (66.5%) | 09 (9.2) |
| 18 | 23 | 21 | 7.8 (94.7%) | 19 (8.2) | 23 | 7.8 (94.1%) | 07 (8.2) | 21 | 7.5 (94.5%) | 07 (7.9) |
| 19 | 19 | 16 | 7.6 (93.0%) | 20 (8.1) | 19 | 8.9 (93.7%) | 13 (9.5) | 18 | 7.6 (89.0%) | 13 (8.5) |
| 20 | 20 | 18 | 5.2 (52.6%) | 10 (9.8) | 20 | 6.0 (55.3%) | 03 (10.8) | 18 | 5.0 (53.0%) | 03 (9.4) |
| **Total** | 496 | 469 | 188.3 (88.6%) | (212.5) | 475 | 207.4 (86.9%) | (238.6) | 468 | 188.1 (88.9%) | (211.5) |

1. Cover length was the physic length in which the two EST-SSR covered region in physic map, the blanket data represented ratio between cover length and corresponding physic length .

b.The blanket data in **Chrs** column represented the total physic length of corresponding chromosome.
