# Supplementary material for: A High-Density EST-SSR-Based Genetic Map and QTL Analysis of Dwarf Trait in Cucurbita pepo L
Source: Int J Mol Sci. 2018 Oct 12;19(10):3140. doi: 10.3390/ijms19103140 (PMC6213718; doi:10.3390/ijms19103140)
Supplement: Supplementary file 1 [file ijms-19-03140-s001.zip › Supplementary Files/Table S5.DOCX]

**Table S5. QTL analysis for dwarf trait at young and mature developmental stage in *C. pepo***

| **Trait** | **QTL^a^** | **LG** | **Position (cM)** | **Flanking markers** | **LOD** | **Add^b^** | **Dom^c^** | **PVE (%)^d^** |
| --- | --- | --- | --- | --- | --- | --- | --- | --- |
| *Dwarf* | *qCpDy1* | 20 | 2.0 | **CMTp36** -- PU051973 | 42.58 | -10.50 | -8.25 | 55.1 |
|  | *qCpDy2* | 20 | 22.0 | PU092399 -- PU012717 | 6.66 | 3.46 | 3.04 | 5.8 |
|  | *qCpDm1* | 20 | 0.0 | **CMTp36** – PU001573 | 29.71 | -21.3 | -0.1 | 45.7 |
|  | *qCpDm2* | 20 | 5.0 | PU075627 – CMTm61 | 5.21 | -0.1 | -10.5 | 5.97 |
|  | *qCpDm3* | 15 | 45.0 | PU036119 – CMTmC12 | 4.47 | -8.1 | -5.1 | 6.6 |

^a^ qCpDy and qCpDm, QTL for Dwarf at young and mature stage in *C. pepo*; ^b^ Add, Additive effect;  ^c^ Dom, Dominant effects; ^d^ PVE, Phenotypic variance explained.
