# Supplementary material for: A High-Density EST-SSR-Based Genetic Map and QTL Analysis of Dwarf Trait in Cucurbita pepo L
Source: Int J Mol Sci. 2018 Oct 12;19(10):3140. doi: 10.3390/ijms19103140 (PMC6213718; doi:10.3390/ijms19103140)
Supplement: Supplementary file 1 [file ijms-19-03140-s001.zip › Supplementary Files/Figure S1.doc]

**Figure S1. *C. pepo* parents used for map construction.**

Two inbred lines differing in plant architecture**(A)**, internode length**(B)**, young female formation**(C)**, leaf shape**(D)**, fruit shape and color**(E)**, seed dimention **(F)** and mesocarp color **(G)**. From (A) to (G), left is vein parent "Jinganlu" (pumpkin type), and right is the dwarf parent "HM-S2" (zucchini type). Bars=5 cm.
