# Supplementary material for: A High-Density EST-SSR-Based Genetic Map and QTL Analysis of Dwarf Trait in Cucurbita pepo L
Source: Int J Mol Sci. 2018 Oct 12;19(10):3140. doi: 10.3390/ijms19103140 (PMC6213718; doi:10.3390/ijms19103140)
Supplement: Supplementary file 1 [file ijms-19-03140-s001.zip › Supplementary Files/Figure S2.doc]

**Figure S2. Collinearity with two previous gSSR genetic map.**

Map comparison between genetic map in this research (LGs) and two previous gSSR maps (LGps for *C. pepo* and LGms for *C. moschata*) based on common markers (connected by broken lines). Marker distance are given in cM. EST-SSR in LGs were represented by blue line, gSSR in LGs were represented by red line and common gSSR were highlighted by bold. LGps with common markers with different LGs were highlighted with same color.
