# Supplementary material for: A High-Density EST-SSR-Based Genetic Map and QTL Analysis of Dwarf Trait in Cucurbita pepo L
Source: Int J Mol Sci. 2018 Oct 12;19(10):3140. doi: 10.3390/ijms19103140 (PMC6213718; doi:10.3390/ijms19103140)
Supplement: Supplementary file 1 [file ijms-19-03140-s001.zip › Supplementary Files/Figure S3.doc]

**Figure S3. Collinearity between genetic map(blue) and physic maps(gray).**

**(A)** Collinearity for *C. moschata* genome. **(B)** Collinearity for *C. maxima* genome.
