# Supplementary material for: A High-Density EST-SSR-Based Genetic Map and QTL Analysis of Dwarf Trait in Cucurbita pepo L
Source: Int J Mol Sci. 2018 Oct 12;19(10):3140. doi: 10.3390/ijms19103140 (PMC6213718; doi:10.3390/ijms19103140)
Supplement: Supplementary file 1 [file ijms-19-03140-s001.zip › Supplementary Files/Figure S4.doc]

**Figure S4. Plant height and comparation QTLs of dwarf trait in different reports and hormone response.**

**(A) and (B)** Internode number (A) and average internode length (B) at young and mature stage. Data from three environment (BJA, LFA and LFS) were analyzed, bars show mean ± SD (n=45). Different letters indicates significant difference at *P＜0.05*.

**(C)** Comparison major QTL position for dwarf trait in this research (***qCpDy1*** and ***qCpDm1***) and two previous research (***qCmB2*** in *C. maxima* and ***B*** in *C. pepo*). Hits between LG20 and Cp4.1LG10, *Cma_Chr03* were represented by gray lines. The two candidate gene GA20ox and GA2ox and their physic position in chromosomes were marked. The gene ***qCmB2*** which designated as a GA20ox in previous research was also marked.

**(D)** Responses of dwarf parent to different concentrations of Gibberellins (GA) and vein parent to different concentrations of Paclobutrazol (PAC). Exogenous hormone concentration was represented by logarithms of concentration (Log M), bar show mean ± SE (n=15).
