# Supplementary material for: A High-Density EST-SSR-Based Genetic Map and QTL Analysis of Dwarf Trait in Cucurbita pepo L
Source: Int J Mol Sci. 2018 Oct 12;19(10):3140. doi: 10.3390/ijms19103140 (PMC6213718; doi:10.3390/ijms19103140)
Supplement: Supplementary file 1 [file ijms-19-03140-s001.zip › Supplementary Files/Figure S5.doc]

**Figure S5. Aligment results between *C. pepo* and melon and cucumber genomes.**

**(A) and (B)** Dot plots represent the aligment results between *C. pepo* and melon (A) and cucumber genomes(B). The horizontal axis shows the genetic position of 20 LGs of *C. pepo*, while the vertical axis shows the physical position of the 12 melon (A) and 7 cucumber (B) chromosomes. Each dot corresponding to a single marker represents one tBLASTx hit between *C. pepo* LGs and melon and cucumber chromosomes.

**(C) and (D)**Three categories reveal pairwise relationships among 20 linkage groups. In this, **Category A** includes twelve LGs (LG20 and LG18, LG5 and LG14, LG16 and LG12, LG17 and LG3, LG2 and LG6, and LG7 and LG9) that show roughly two-to-one relationships with chromosomes of melon (C) and cucumber (D). **Category B** includes three LGs (LG4, LG8, and LG15). The front region of LG4 corresponds with LG8 while the backend region of LG4 corresponds to LG15. No syntenic region is shared between LG8 and LG15 in the three genomes. **Category C** includes five LGs (LG1, LG10, LG11, LG13, and LG19).
