# Supplementary material for: A High-Density EST-SSR-Based Genetic Map and QTL Analysis of Dwarf Trait in Cucurbita pepo L
Source: Int J Mol Sci. 2018 Oct 12;19(10):3140. doi: 10.3390/ijms19103140 (PMC6213718; doi:10.3390/ijms19103140)
Supplement: Supplementary file 1 [file ijms-19-03140-s001.zip › Supplementary Files/Table S4.doc]

**Supplementary Table S4. Frequency distribution for average internode length at mature stage in *C. pepo***

| **Population^a^** | **Generation** | **Average internode length (cm)** | | | | | | |
| --- | --- | --- | --- | --- | --- | --- | --- | --- |
|  |  | **＜2** | **2 - 4** | **4 - 6** | **6 - 8** | **8 - 10** | **10 - 12** | **＞12** |
| BJA | P_1_ |  |  |  |  |  |  | 15 |
|  | P_2_ |  | 4 | 11 |  |  |  |  |
|  | F_1_ |  |  | 9 | 5 | 1 |  |  |
|  | BC_1_P_1_ |  |  | 3 | 20 | 7 | 8 | 40 |
|  | BC_1_P_2_ | 5 | 33 | 24 | 4 | 2 |  |  |
|  | F_2_ | 2 | 35 | 45 | 33 | 23 | 13 | 27 |
| LFA | P_1_ |  |  |  |  |  |  | 15 |
|  | P_2_ |  | 5 | 10 |  |  |  |  |
|  | F_1_ |  |  | 8 | 6 | 1 |  |  |
| LFS | P_1_ |  |  |  |  |  |  | 15 |
|  | P_2_ |  | 3 | 12 |  |  |  |  |
|  | F_1_ |  |  | 7 | 7 | 1 |  |  |
|  | F_2_ | 1 | 12 | 16 | 28 | 10 | 4 | 10 |

a. BJA indicates the plants grown in Beijing in autumn 2012; LFA indicates the plants grown in Langfang in autumn 2014; LFS indicates plants grown in Langfang in summer 2015.
